# An Intersectional Approach to Understanding the Psychological Health Effects of Combining Work and Caregiving

**DOI:** 10.1093/geroni/igaf122.427

**Published:** 2025-12-31

**Authors:** Samantha Brady, Taylor Patskanick, Joseph Coughlin

**Affiliations:** Massachusetts Institute of Technology, Cambridge, Massachusetts, United States; Massachusetts Institute of Technology AgeLab, Cambridge, Massachusetts, United States; Massachusetts Institute of Technology AgeLab, Cambridge, Massachusetts, United States

## Abstract

Role theory suggests occupying simultaneous family caregiving and employment roles in midlife may exert positive and negative effects on psychological health. However, there is a lack of causal evidence examining the degree to which combinations of these roles influence psychological health at the intersection of gender and racial identity. Longitudinal data from the Health and Retirement Study (2004–2018) are used to estimate a series of individual fixed effects models examining combinations of employment status and parental caregiving situation on Center for Epidemiological Studies—Depression Scale (CES-D) depression scores among Black and White men and women aged 50–65. Subsequent models were stratified by intensity of caregiving situation and work schedule. Individual fixed effects models demonstrate combining work, and parental caregiving is associated with greater depressive symp¬toms than only working, and with lower depressive symptoms than only caregiving, suggesting that paid employment exerts a protective effect on psychological health whereas parental caregiving may be a risk factor for depressive symptoms in later life. Analyses using an intersectional lens found that combining paid work with parental caregiving exerted a protective effect on CES-D scores among White women and men regardless of participants’ intensity of care situation or work schedule. This effect was not present for Black men and women. Accounting for intersectionality is imperative to research on family caregiving, work, and psychological health.

